# The Outlawry of Disease

**Published:** 1923-12

**Authors:** 


### THE OUTLAWRY OF DISEASE.

### CONTROLLING CONTAGION.

THE ninth annual report, covering the year 1922, of the International Health Board formed under the auspices of the great Rockefeller Foundation, contains a large amount of matter of the utmost interest to sanitarians and others concerned with the elimination of disease and the control of infection. As is invariably the case with everything connected with the Rockefeller Foundation, the report is admirably and lucidly written, and the story it has to tell is in the highest degree encouraging.

### Principles and Practice.

Thus we are told, quite simply, that since the formation of the Board, hook-worm infection the world over has been measurably diminished ; progress has been made towards reducing the ravages of malaria ; and a relentless war is still being waged against yellow fever wherever its danger flag appears :—

From the outset, however, the Board has maintained the conviction that public health is essentially a function of government. No private and temporary agency, whatever its resources, could or should discharge responsibilities which, by their nature, belong to the const:tuted authorities of the commonwealth. Private enterprise, therefore, may be best employed in awakening public opinion and thereby encouraging state and county officials to establish permanent agencies for public health work. Responsibility for the control and cure of any one disease has never been assumed by the Board ; but aid has been given in control and cure where such steps might be expected to demonstrate a need and suggest a possible programme. Lastly, it has been clearly recognised that continued advance in preventive medicine the world over depends upon an adequate supply of skilled public health servants.

### Mastering Yellow Fever and Malaria.

Twenty-five years ago yellow fever menaced virtually the whole of the Western hemisphere :—

At the end of 1922, the only infected areas remaining in the Western Hemisphere appeared to be eastern Mexico and a narrow coastal zone in eastern Brazil from Ceará to Bahia. No other infectious disease has been so completely subjected to human control as has yellow fever, since the day when the Stegomyia was found to be its intermediary host. . . . Dr. Taylor, of Pamlico County, North Carolina, found that administration of quinine in a selected area where local conditions made other measures impracticable, brought about a reduction of malaria of 80.0 per cent. among the white population, and 66.5 per cent. among the coloured.

### Hookworm and Public Health.

Upon the relation between the reduction of hookworm disease and the spread of knowledge of public health, the Report makes this significant comment :—

The relief and control of this disease is a striking object lesson in the control of disease in general. In its nature, causes, and cure, it is easily understood by the common man, and its effects upon his own health and the health of the community are plainly demonstrable. When he has seen this one disease treated and dramatically brought under control, he is prepared to give heed and support to the control of diseases that are less simple and less tangible. . . . Out of forty million people living in Madras Presidency alone, it is estimated that more than thirty-six million are infected with hookworm disease A widespread campaign of popular education is under way, and hospitals are incidentally giving treatment for the disease to all patients as a routine measure. A convincing experiment was undertaken among 298 students of the Madras Medical College who were sceptical of the prevalence of the disease, but were willing to be examined. Two hundred and forty-one were found infected ; and it is safe to assume that as many qualified doctors, with improved health, will advocate hookworm control in those districts in which they undertake private practice.

### Fish Control of Mosquitoes.

In the United States the minnow is being extensively used to eat up the larvæ of the mosquito :—

The value of the top minnow (*Gambusia affinis*) in the control of Anopheles breeding has now been thoroughly demonstrated in many towns throughout the South. Through the county-wide malaria programmes fish control is being rapidly extended. During the year many counties have established hatcheries or largely increased the number already in use. The practice has developed of selecting for this purpose suitable ponds located not only at convenient distributing points, but also near the main highways. Conspicuous signs call the attention of the public to the supply of fish and urge the stocking of all ponds, streams, and other breeding-places. Each body of water stocked usually serves effectively as an additional hatchery. In some places it has been found necessary to use exhibits of Gambusia to instruct the public and especially fishermen to protect them. Boy scouts are often very -ffective in protecting the minnows and stocking numerous minor breeding-places.

